# Determinants of non-use of antenatal care services in eastern Indonesia: analysis of the 2023 Indonesia health survey

**DOI:** 10.3389/fgwh.2025.1649276

**Published:** 2025-08-18

**Authors:** Christiana Rialine Titaley, Dwi Hapsari Tjandrarini, Maxwell Landri Vers Malakauseya, Iwan Ariawan, Ressita Fannia Iwan, Sean Samuel Istia, Michael J. Dibley

**Affiliations:** ^1^ Department of Public Health, Faculty of Medicine Pattimura University, Ambon, Indonesia; ^2^ National Research and Innovation Agency Republic of Indonesia, Bogor, Indonesia; ^3^ Faculty of Medicine, Pattimura University, Ambon, Indonesia; ^4^ Faculty of Public Health, Universitas Indonesia, Depok, Indonesia; ^5^ Sydney School of Public Health, Faculty of Medicine and Health, Sydney, NSW, Australia

**Keywords:** antenatal care services, maternal health, health care utilization, public health, pregnancy, community health

## Abstract

**Introduction:**

Although Indonesia has made significant progress in improving maternal and child health nationally, regional disparities persist, particularly in eastern Indonesia, where maternal and neonatal health outcomes remain suboptimal compared with the western regions. This study examined factors associated with non-use of antenatal care (ANC) in eastern Indonesia.

**Methods:**

We analyzed data from 3,261 mothers with infants under one year of age in eastern Indonesia who were interviewed in the 2023 Indonesia Health Survey. The primary outcome was maternal non-use at ANC during pregnancy with an infant younger than 12 months at the time of the survey. Eighteen potential predictors of non-use of ANC were assessed using a multilevel analysis.

**Results:**

Approximately 5% (95% CI: 4.14–6.09) of the mothers with infants 0–11 months did not seek antenatal care. Non-use was associated with infant age, region, socioeconomic status, health checks, knowledge of stunting, and pregnancy-related complications. Mothers with infants aged 6–11 months were 63% less likely to forgo ANC [adjusted odds ratio (aOR) = 0.63, *p* *=* *0.049*]. Living in Sulawesi (aOR = 2.66, *p* *=* *0.001*), Maluku (aOR = 13.76, *p* *<* *0.001*), and Papua (aOR = 17.72, *p* *<* *0.001*) increased ANC non-use. The poorest households had 9.90 times higher odds of non-use than the richest households (*p* *<* *0.001*). Higher non-use was also linked to no prior health checks (aOR = 2.54, *p* *=* *0.006*), low stunting knowledge (aOR = 2.93, *p* *=* *0.004*), and no pregnancy complications (aOR = 4.30, *p* *=* *0.001*).

**Conclusions:**

Socioeconomic and geographic disparities drive non-use of antenatal care in eastern Indonesia. Improving healthcare access, education, and early screening are crucial for reducing regional inequalities and enhancing maternal health.

## Introduction

Antenatal care (ANC) has been widely recognized as a key component of reproductive health services that provides a platform for essential healthcare functions to ensure the well-being of both mother and child during pregnancy (1). Regular ANC visits offer a wide range of essential services for pregnant women, including early detection and management of pregnancy-related complications, nutritional support, immunization, and counselling on safe delivery and new-born care (1, 2). Studies showed that the lack of ANC was associated with an increased risk of maternal and neonatal complications that could lead to various adverse outcomes, such as preterm birth, low birth weight, stillbirth, and even neonatal and maternal mortality (3–5).

The World Health Organization (WHO) recommends a minimum of eight ANC visits during pregnancy to optimize maternal and foetal health outcomes (1). Nevertheless, in many low—and middle-income countries, ANC utilization remains suboptimal, with sometimes disparities across regions, particularly in remote and underdeveloped areas where barriers such as limited health infrastructure and geographic inaccessibility usually affect the uptake of the service (6).

The Ministry of Health (MOH) in Indonesia recommends that pregnant women attend at least six antenatal visits during pregnancy: one in the first trimester, two in the second trimester, and three in the third trimester. Although this is fewer than the eight-contact model recommended by the WHO (7), the national guideline represents a progressive shift from the previous standard of four visits, aiming to improve maternal and neonatal outcomes within the context of Indonesia's healthcare capacity and regional disparities. This adaptation balances global recommendations with local feasibility and accessibility constraints, particularly in remote and underdeveloped areas. National data also showed a steady increase in the proportion of women accessing ANC services over time as the proportion of pregnant women receiving at least one ANC visit increased from 95.4% in 2013 (8) to 96.6% in 2023 (9).

However, the national reports showed that the proportion varied considerably across regions (2, 10). The latest 2023 Indonesia Health Survey reported that the proportion of women attending at least one ANC visit in eastern Indonesia was much lower than in western Indonesia. For example, while 99.6% of pregnant women in Bali (Western Indonesia) received at least one ANC visit, only 58.7% of pregnant women in the Papua region (Eastern Indonesia) did so (9). Studies also reported a higher mortality rates including perinatal and infant mortality in the eastern than western region of Indonesia (4, 11). Studies showed that these deaths was also found to be associated with inadequate antenatal care (11, 12). This evidence indicates that despite the significant progress made by the Government of Indonesia to improve maternal and child health status nationally over the past few decades (9, 13), the outcomes remain suboptimal in eastern Indonesia (4, 14).

Numerous studies have reported the role of various factors influencing ANC use, such as maternal knowledge (14), wealth index (2, 4), and access to healthcare facilities (15, 16). Nevertheless, specific variations in healthcare-seeking behavior in eastern Indonesia remain underexplored. Addressing this gap is therefore important for policymakers and program managers to understand the region-specific determinants of ANC use and further design targeted, region-specific interventions.

In 2023, the MOH of the Republic of Indonesia conducted the 2023 Indonesian Health Survey, which assessed key health indicators nationwide (9). This survey provides essential baseline data on maternal and child health, nutrition, and healthcare use to inform national health policies. Using data from this survey, this analysis aimed to identify factors associated with the non-use of ANC in eastern Indonesia. These findings will help inform strategies to enhance ANC coverage and ultimately reduce maternal and neonatal morbidity and mortality in eastern Indonesia and Indonesia in general.

## Materials and methods

### Data source and survey design

The data used in this analysis were derived from the 2023 Indonesian Health Survey (9), conducted by the MOH, Republic of Indonesia, using a multistage systematic random sampling method. Since 2007, the MOH has conducted a cross-sectional household health survey every five years to establish baseline data and monitor health-related indicators at the district, provincial, and national levels. The 2023 survey sample size was designed to allow for district-level estimations (9). A detailed explanation of the survey methodology has been provided elsewhere (9).

The 2023 Indonesian Health Survey included a representative sample of households from 38 provinces and 514 districts or cities. It covered 345,000 general households for the basic health survey and an additional 104,000 households with children under five years of age for the nutritional survey. These households were selected from 34,500 census blocks.

**Figure 1:**
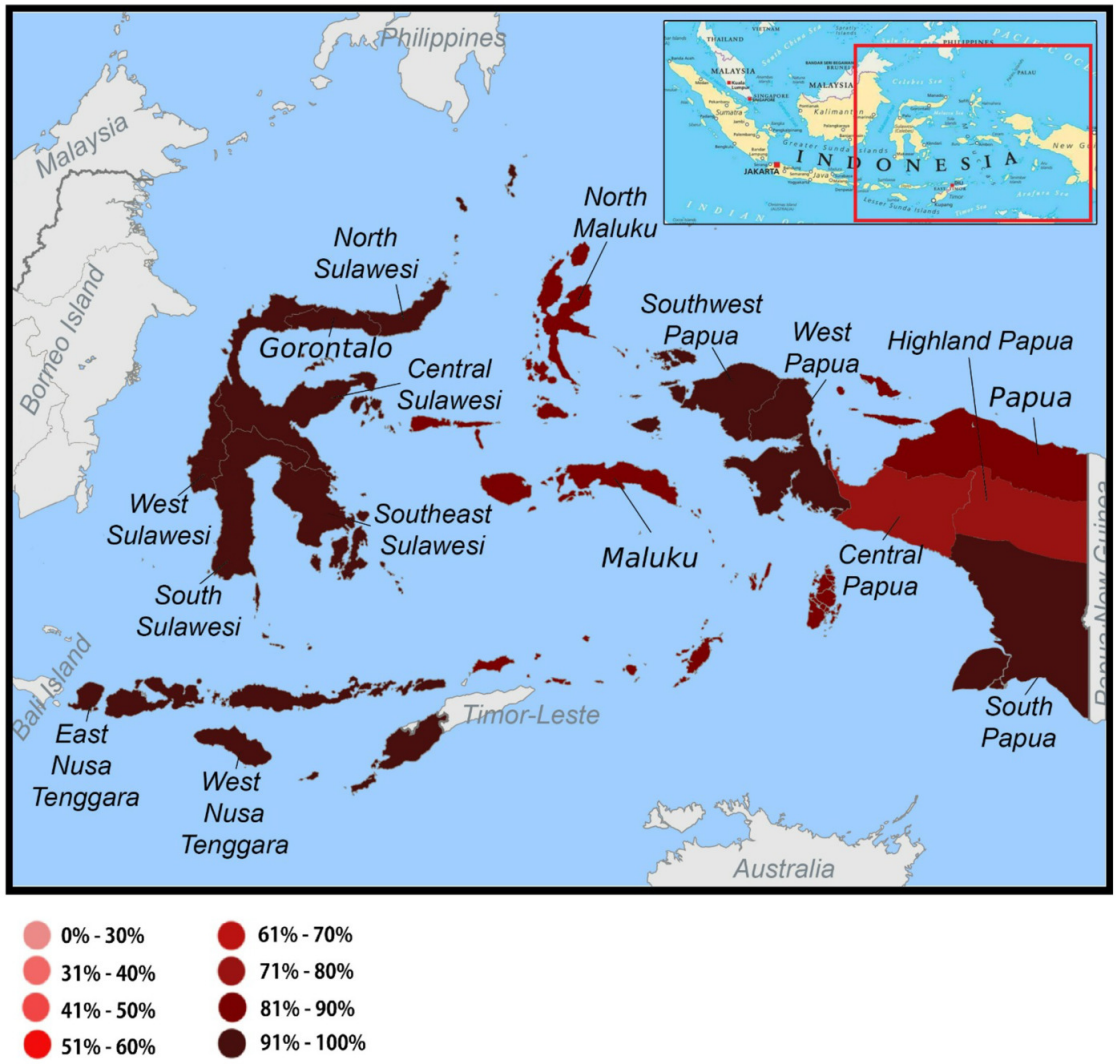
Distribution of the non-use of antenatal care service among mothers with infants aged 0–11 months in the eastern part of Indonesia, the 2023 Indonesia health survey.

At the individual level, 1,191,692 participants were interviewed. For this analysis, we used data from 3,261 mothers with infants aged 0–11 months residing in eastern Indonesia. The region classified as eastern Indonesia comprises Nusa Tenggara Barat, Nusa Tenggara Timur, Sulawesi, Maluku, and Papua (Figure 1). The data were accessed on 1 December 2024, for the purposes of the current research.

### Outcome variable

The primary outcome was maternal non-useuse at ANC during the pregnancy in infants younger than 12 months at the time of the survey. The non-use was determined based on the question: “Did you seek ANC from a healthcare provider?” Mothers who did not seek ANC services were assigned a score of 1, while those who attended ANC were assigned a score of 0.

### Potential predictors

We included 18 potential predictors of ANC non-use, categorized into five main groups: community factors, household characteristics, parental demographics, maternal exposure to health information and healthcare, and maternal obstetric characteristics. We included the child's age at the time of the survey to account for potential recall bias since mothers of older infants may recall ANC visits less accurately, and to account for contextual differences that may influence ANC use.

Two variables were included in the community characteristics: region (West Nusa Tenggara, East Nusa Tenggara, Sulawesi, Maluku, and Papua) and type of residence (urban or rural). In terms of household characteristics, the number of children under five years old (one, two, or three or more) and the household wealth index (richest, rich, middle, poor, poorest) were included. The household wealth index was constructed using Principal Component Analysis16 based on 16 housing characteristics and assets owned by respondents: (1) predominant wall material, (2) predominant floor material, (3) source of drinking water, (4) type of latrine, (5) method of waste disposal, (6) source of electricity, (7) type of cooking fuel, (8) ownership of a washing machine, (9) ownership of a refrigerator, (10) ownership of a mobile phone, (11) ownership of an air conditioner, (12) ownership of a computer, (13) ownership of a flat-screen television (minimum 30 inches), (14) ownership of a motorcycle, (15) ownership of a car, and (16) ownership of at least 10 grams of gold.

For the parental demographic characteristics, we considered four variables: maternal education (academy or university, completed senior high school, completed junior high school, incomplete elementary school), maternal occupation (housewife or not working outside the home, formal worker, informal worker, or student), paternal education (academy or university, completed senior high school, completed junior high school, incomplete elementary school), and paternal occupation (formal worker, informal worker, or unemployed).

Three variables were included in the group of maternal exposure to health information and care: health-seeking behavior (once or more every six months, once a year or less, never), knowledge and awareness about pregnancy danger signs (high, low), and knowledge and awareness about stunting (high, low). To calculate the respondents' level of knowledge about the danger signs of pregnancy, we used the information from seven questions. These included signs such as vaginal bleeding, swelling of the legs, hands, or face accompanied by headache, high fever, convulsions, early rupture of membranes, reduced or no fetal movement, and persistent vomiting or loss of appetite. The correct response for each question was assigned a score of one. The total knowledge score was calculated, and those scoring above the median were classified as having a high level of knowledge, whereas those scoring at or below the median were categorized as having a low level of knowledge.

The knowledge and awareness of stunting was assessed based on four knowledge groups: definition (nine correct answers), causes (six correct answers), impact (six correct answers), and prevention (eight correct answers) of stunting. The total knowledge score ranged from 0 (all responses were incorrect) to 29 (all correct). Respondents with scores above the median were classified as having a high level of knowledge, whereas those with scores at or below the median were categorized as having a low level of knowledge.

Finally, we included six variables for maternal exposure to health information and care: age at childbirth (<20, 20–29, 30–39, ≥40 years), pregnancy order (first pregnancy, second or third pregnancy, and fourth or more), number of childbirths, history of abortion (yes/no), presence of pregnancy complications (yes or no), and pregnancy intention (yes, no, or only then).

### Data analysis

First, descriptive statistical methods were used to examine the distribution of all variables included in the analysis. This was followed by bivariate analyses to explore the distribution of these variables according to ANC attendance status. We then used multilevel analysis with two sequential models that incorporated random intercepts to examine the factors associated with the non-use of ANC.

In the multilevel analysis, a three-level logistic regression model was applied to account for the hierarchical structure of the data, with individuals (Level 1) nested within Primary Sampling Units (PSUs) (Level 2), which were further nested within districts (Level 3) and provinces (Level 4). The null model (also called an empty model), which included no explanatory variables, was constructed to examine the extent to which the primary sampling unit, district, and province levels were associated with ANC attendance without adjusting for individual, household, or community-level predictors. The median odds ratio (MOR) was calculated for each level to quantify its contribution to ANC nonattendance.

Following the null model, we constructed Model 1 by introducing all the explanatory variables. This allowed us to assess the extent to which the explanatory characteristics explained the variation in ANC non-use while accounting for the nested structure of the data. We applied backward elimination to remove factors that were not significantly associated with the outcome, using a significance threshold of 0.05. Four variables— the child's age, region, type of residence (urban or rural), and household wealth index—were selected *a priori* and retained in the final model regardless of their statistical significance. Adjusted odds ratios (aORs) were used to estimate the association between each potential predictor and the study outcome.

The final model presented adjusted odds ratios (aORs) and 95% confidence intervals (95% CIs) for all predictors. This analysis accounted for the complexity of the sample design. Multilevel models were developed using the Stata/MP software (version 14.2; StataCorp, College Station, TX, USA) with a melogit routine.

### Ethical approval and informed consent

This study was conducted in accordance with the ethical principles outlined in the Declaration of Helsinki. Ethical approval for the 2023 Indonesia Health Survey was obtained from the Health Research Ethics Committee of the Health Polytechnic of the Ministry of Health Jakarta II (Komisi Etik Penelitian Kesehatan Poltekkes Kemenkes Jakarta II), under reference number LB.02.01/I/KE/L/287/2023.

## Results

This analysis used information collected from 3,262 mothers of infants aged 0–11 months residing in eastern Indonesia. Most mothers were from households with only one child under five years of age (68.0%) and had a partner who worked in the informal sector (77.4%). Most mothers were housewives (61.1%), had a low level of awareness of stunting (77.6%), and did not experience any pregnancy complications (83.9%) (Table 1).

**Table 1:** Frequency distribution of factors analyzed in this study, the 2023 Indonesia health survey.

Variable	*n*	%	Never had ANC		Ever had ANC	
			*n*	%	*n*	%
Child's age (months)						
<6	1,605	49.2	89	5.5	1,517	94.5
6-<12	1,656	50.8	75	4.6	1,581	95.5
Community level						
Region						
NTB	480	14.7	5	1.1	475	98.9
NTT	451	13.8	12	2.7	439	97.3
Sulawesi	1,762	54.0	53	3.0	1,708	97.0
Maluku	241	7.4	33	13.8	208	86.2
Papua	328	10.1	60	18.2	268	81.8
Type of residence						
Urban	1,382	42.4	50	3.6	1,332	96.4
Rural	1,879	57.6	114	6.1	1,765	93.9
Household level						
Number of children under five living in the household						
One	2,219	68.0	100	4.5	2,119	95.5
Two	961	29.5	57	5.9	905	94.1
Three or more	81	2.5	8	9.4	74	90.6
Household wealth index						
Richest	691	21.2	13	1.8	679	98.2
Rich	738	22.6	26	3.5	713	96.5
Middle	665	20.4	18	2.7	647	97.3
Poor	653	20.0	27	4.2	625	95.8
Poorest	514	15.8	81	15.7	433	84.3
Parental demographic characteristics						
Maternal education						
Academy/university	699	21.4	21	2.9	678	97.1
Completed senior high school	1,217	37.3	50	4.1	1,167	95.9
Complete junior high school	543	16.6	26	4.8	516	95.2
Incomplete elementary school	803	24.6	67	8.3	736	91.7
Maternal occupation						
Housewife/not working outside	1,992	61.1	101	5.1	1,891	94.9
Formal workers	332	10.2	15	4.4	317	95.6
Informal workers/students	938	28.8	48	5.2	889	94.8
Paternal education						
Academy/university	621	19.0	29	4.7	592	95.3
Completed senior high school	1,410	43.2	57	4.1	1,353	96.0
Complete junior high school	462	14.2	29	6.4	432	93.6
Incomplete elementary school	769	23.6	48	6.3	720	93.7
Paternal occupation						
Formal worker	656	20.1	40	*6*.*2*	616	93.8
Informal worker	2,524	77.4	115	*4*.*5*	2,410	95.5
Unemployed	81	2.5	9	*10*.*9*	72	89.2
Maternal exposure to health information and care						
Health seeking behavior						
Once or more every 6 months	2,078	63.7	63	3.0	2,016	97.0
Once a year or less	467	14.3	32	6.9	435	93.1
Never	716	22.0	69	9.6	647	90.4
Knowledge about the danger sign of pregnancy						
High	1,077	33.0	44	4.1	1,033	95.9
Low	2,185	67.0	120	5.5	2,065	94.5
Knowledge about stunting						
High	732	22.4	12	1.6	720	98.4
Low	2,530	77.6	152	6.0	2,378	94.0
Maternal obstetric characteristics						
Age at childbirth (years old)						
20–29	1,388	42.6	65	4.7	1,323	95.3
<20	119	3.7	10	8.4	109	91.6
30–39	1,484	45.5	68	4.6	1,416	95.4
≥40	271	8.3	21	7.7	250	92.3
Rank of pregnancy						
1st pregnancy	565	17.3	33	5.9	531	94.1
2nd/3rd pregnancy	1,889	57.9	79	4.2	1,810	95.8
4th or more pregnancy	808	24.8	52	6.4	756	93.6
Number of child-birth						
1st delivery	642	19.7	37	5.8	605	94.2
2nd/3rd delivery	1,956	60.0	83	4.3	1,872	95.8
4th or more delivery	664	20.4	44	6.6	620	93.4
History of abortion before						
Yes	97	3.0	8	8.0	89	92.0
No	3,165	97.0	156	4.9	3,009	95.1
Presence of pregnancy complication						
Yes	525	16.1	5	1.0	520	99.0
No	2,736	83.9	158	5.8	2,578	94.2
Desire for pregnancy						
Yes	3,029	92.9	155	5.1	2,873	94.9
No/only then	233	7.1	9	3.8	224	96.2

**Figure 2:**
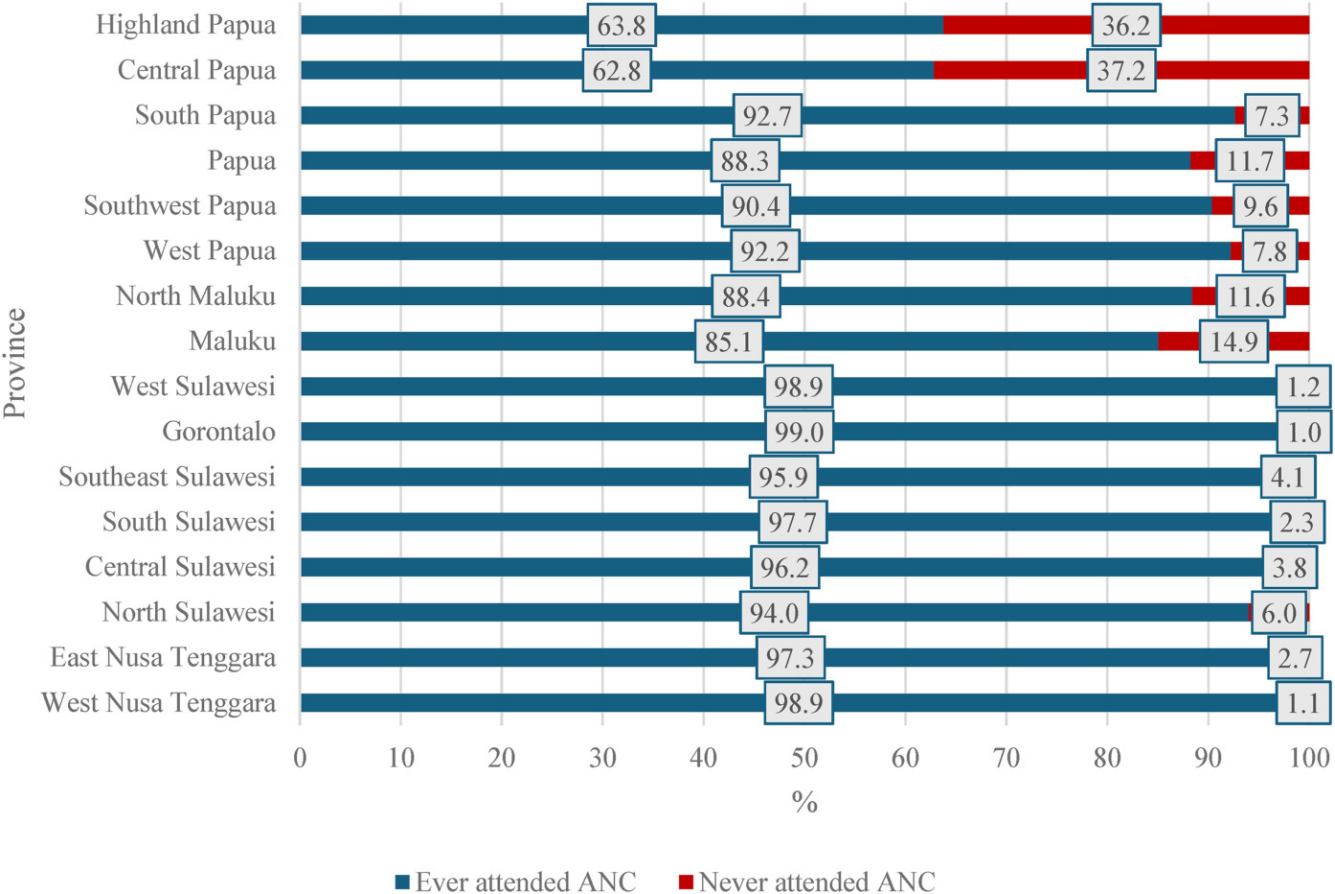
Frequency distribution of antenatal care use by mothers with infants aged 0–11 months in the eastern part of Indonesia, the 2023 Indonesia health survey.

Our analysis showed that approximately 5% (95% CI: 4.14–6.09) of the mothers did not seek antenatal care services from healthcare providers. The province of Central Papua had the highest percentage of mothers not attending ANC services (37.2%, 95% CI: 22.9–54.2) (Figure 2). The proportion of ANC non-use by district in each region is presented in Supplementary Figures S1–S5.

Table 2 shows the multilevel modelling results of the factors associated with the non-use of ANC services. Interestingly, mothers with infants aged 6 to 12 months at the time of the interview were more likely to have attended ANC services during their pregnancy than mothers with younger infants (aged <6 months). This was mirrored by the reduced odds of not attending ANC among mothers of infants aged 6 to <12 months (aOR = 0.63, 95% CI: 0.40–1.00, *p* = 0.049). Furthermore, mothers residing in Sulawesi (aOR = 2.66, 95% CI: 1.49–4.75, *p* = 0.001), Maluku (aOR = 13.76 95% CI: 5.79–54.29, *p* < 0.001), and Papua (aOR = 17.72, 95% CI: 5.79–54.29, *p* < 0.001) demonstrated significantly higher odds of not attending ANC services compared to those living in Nusa Tenggara Barat region.

**Table 2:** Factors associated with the non-use of ANC among mothers with children aged 0–11 months living in the eastern region of Indonesia, the 2023 Indonesia health survey.

Variable	Univariable				Null Model				Model 1 Multivariable analysis			
	OR	95% CI		*p value*					OR	95% CI		*p value*
Child's age												
<6 month	1.00								1.00			
6-<12 month	0.67	0.40	1.11	0.121					0.63	0.40	1.00	0.049
Community level												
Region												
NTB	1.00								1.00			
NTT	3.12	2.57	3.78	<0.001					1.04	0.68	1.60	0.845
Sulawesi	3.44	2.01	5.92	<0.001					2.66	1.49	4.75	0.001
Maluku	31.50	15.39	64.48	<0.001					13.76	9.22	20.51	<0.001
Papua	60.84	12.32	300.32	<0.001					17.72	5.79	54.29	<0.001
Type of residence												
Urban	1.00								1.00			
Rural	2.11	1.20	3.71	0.009					1.12	0.73	1.73	0.591
Household level												
Number of children under five living in the household												
One	1.00											
Two	1.56	1.00	2.45	0.052								
Three or more	2.53	0.88	7.26	0.084								
Household wealth index												
Richest	1.00								1.00			
Rich	2.08	0.90	4.79	0.087					1.95	0.82	4.64	0.130
Middle	1.95	0.63	5.98	0.245					1.64	0.48	5.64	0.434
Poor	3.26	1.36	7.79	0.008					2.46	0.92	6.56	0.073
Poorest	15.83	5.00	50.11	<0.001					9.90	3.35	29.31	<0.001
Parental demographic characteristics												
Maternal education												
Academy/university	1.00											
Completed senior high school	1.89	0.89	4.00	0.100								
Complete junior high school	2.54	1.13	5.71	0.023								
Incomplete elementary school	4.90	2.27	10.58	<0.001								
Maternal occupation												
Housewife/not working outside	1.00											
Formal workers	0.60	0.22	1.65	0.322								
Informal workers/students	0.97	0.54	1.76	0.941								
Paternal education												
Academy/university	1.00											
Completed senior high school	1.11	0.47	2.62	0.808								
Complete junior high school	2.23	0.97	5.08	0.057								
Incomplete elementary school	3.20	1.50	6.85	0.003								
Paternal occupation												
Formal worker	1.00											
Informal worker	1.43	0.71	2.88	0.319								
Unemployed	3.23	0.98	10.61	0.054								
Maternal exposure to health information and care												
Health seeking behavior												
Once or more every 6 months	1.00	1.00							1.00			
Once a year or less	2.21	1.07	4.53	0.031					1.70	0.81	3.54	0.158
Never	3.56	1.73	7.35	0.001					2.54	1.31	4.96	0.006
Knowledge about the danger sign of pregnancy												
High	1.00	1.00										
Low	2.66	1.45	4.89	0.002								
Knowledge about stunting												
High	1.00	1.00							1.00			
Low	5.62	2.67	11.84	<0.001					2.93	1.41	6.10	0.004
Maternal obstetric characteristics												
Age at child-birth												
20–29	1.00	1.00										
<20	4.24	1.47	12.19	0.007								
30–39	0.90	0.50	1.63	0.724								
≥40	2.16	0.88	5.32	0.094								
Rank of pregnancy												
1st pregnancy	1.00	1.00										
2nd/3rd pregnancy	0.72	0.35	1.45	0.357								
4th or more pregnancy	1.12	0.57	2.22	0.734								
Number of child-birth												
1st delivery	1.00	1.00										
2nd/3rd delivery	0.72	0.34	1.52	0.394								
4th or more delivery	1.21	0.57	2.58	0.619								
History of abortion before												
Yes	1.00	1.00										
No	0.75	0.17	3.21	0.694								
Presence of pregnancy complication												
Yes	1.00	1.00							1.00			
No	5.91	2.20	15.91	<0.001					4.30	1.75	10.57	0.001
Desire for pregnancy												
Yes	1.00	1.00										
No/only then	0.71	0.20	2.50	0.594								
Province Median Odds Ratio					4.06				1.00			
District Median Odds Ratio					2.61				2.52			
PSU Median Odds Ratio					4.67				5.30			

**Figure 3:**
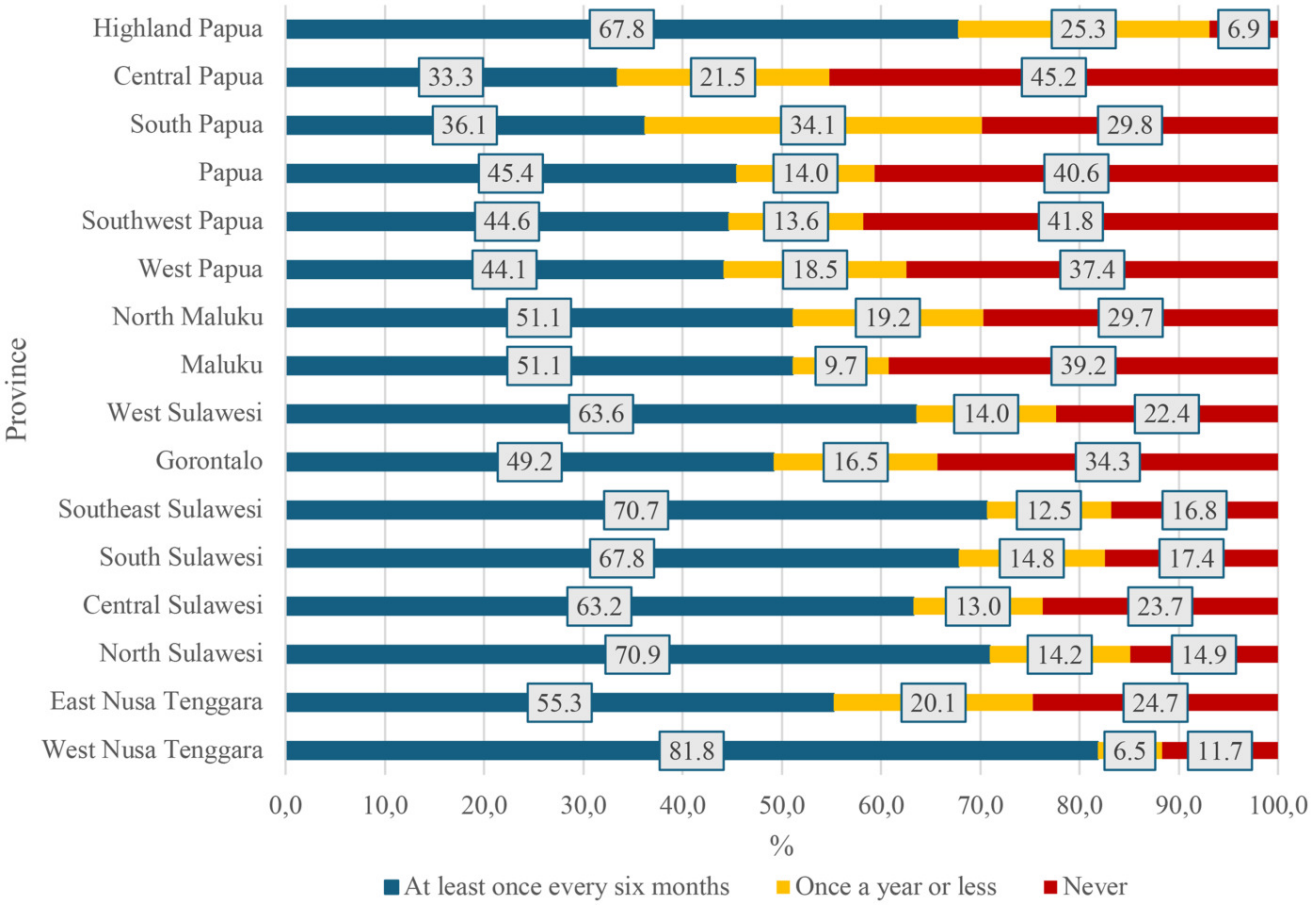
Frequency distribution of general health examination among mothers with infants aged 0–11 months in the eastern part of Indonesia, the 2023 Indonesia health survey.

At the household level, mothers from the poorest households had significantly higher odds of not attending ANC services than those from the richest households (aOR = 9.90, 95% CI: 3.35–29.31, *p* < 0.001). As expected, mothers who had never had any health checks were more likely not to attend ANC services than those who underwent health checks regularly at least once every six months (aOR = 2.54, 95%CI:1.31–4.96, *p* = 0.006). Figure 3 presents the distribution of general health examinations among mothers in eastern Indonesia. The highest percentage of those who had never had any health checks (45.2%) was found in Central Papua Province (95% CI: 29.1–62.3).

**Figure 4:**
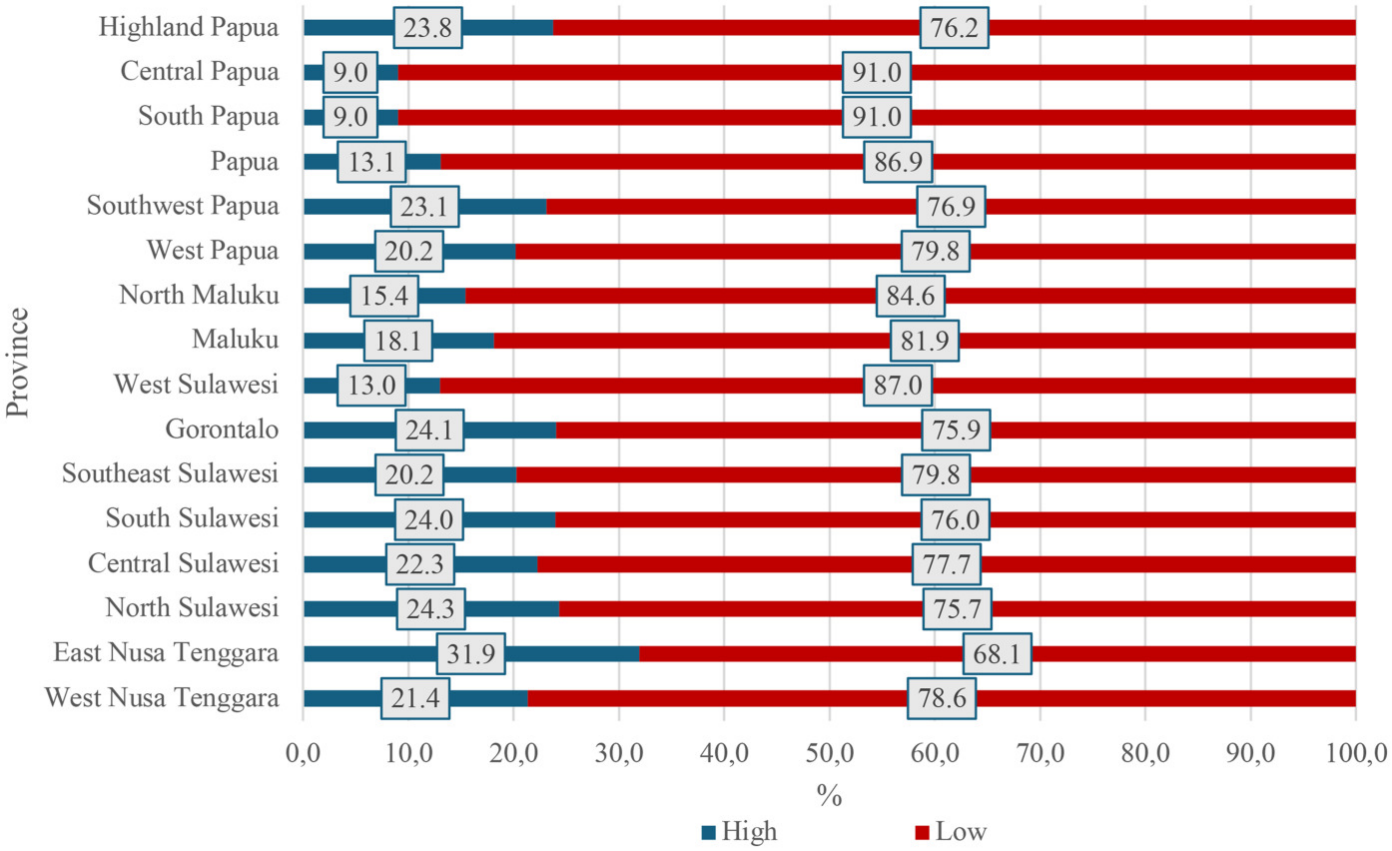
Frequency distribution of the level of knowledge and awareness about stunting among mothers with infants aged 0–11 months in the eastern part of Indonesia, the 2023 Indonesia health survey.

Moreover, our analysis showed that a low level of maternal knowledge of stunting was also associated with mothers’ non-use at ANC services (aOR = 2.93, 95% CI: 1.41–6.10, *p* = 0.004). Notably, Central and South Papua had the highest proportion of mothers with a low level of knowledge and awareness about stunting at 91.0% (Figure 4). In addition, the absence of pregnancy complications was significantly associated with non-use at ANC services (aOR = 4.30, 95% CI: 1.75–10.57, *p* = 0.001).

In the null model, the MOR was 4.06 at the province level, 2.61 at the district level, and 4.67 at the PSU level, indicating substantial variation in ANC non-use across all three geographic levels when no explanatory variables were included. After adjusting for all significant predictors in Model 1, the MOR decreased at the provincial level (to 1.00), suggesting that individual characteristics fully explained the variance between provinces. However, the MOR remained relatively unchanged at the district level (2.52) but increased at the PSU level (5.30), indicating substantial variation in average individual characteristics across PSU.

## Discussion

### Main findings

Our analysis highlighted the role of environmental, household, and maternal factors in ANC non-use among mothers with infants aged 0–11 months residing in eastern Indonesia. ANC attendance was lower among mothers of younger infants (<6 months) than among those of older infants. At the environmental level, the geographic region emerged as a significant predictor of ANC non-use. At the household level, the household wealth index was significantly associated with ANC use, with lower wealth linked to lower service utilization. Among maternal-level factors, limited health-seeking behavior, poor knowledge about stunting, and absence of pregnancy complications were all significantly associated with non-use. These findings underscore the need for targeted, context-specific interventions to improve ANC coverage, especially among the disadvantaged and underserved populations in eastern Indonesia.

### Factors associated with ANC non-use in eastern Indonesia

Our findings showed that ANC attendance was lower among mothers with infants aged <6 months than mothers with older infants. This finding may possibly reflect the evolving impact of the Coronavirus Disease 2019 (COVID-19) pandemic. Although we did not have data on the exact timing of pregnancy in relation to the pandemic phases, it is plausible that heightened concern over COVID-19 in early 2022 encouraged greater ANC engagement as a protective health-seeking behavior (9). However, as fear declined by late 2022, care-seeking behavior also appeared to decrease. The transition from emergency to routine health system operations led to the reallocation of resources, staffing shortages, and lingering healthcare worker burnout, which may have disrupted ANC availability (17, 18). The long-term economic effects of the pandemic likely forced some women to deprioritize healthcare visits due to financial constraints (19).

Our study confirmed regional disparities in ANC utilization. Mothers in the Sulawesi, Maluku, and Papua regions were less likely to attend services than those living in Nusa Tenggara Barat. Several potential barriers hinder ANC attendance in these regions, such as geographic isolation, limited health infrastructure, workforce shortages, cultural beliefs, low health literacy, and economic hardship (14, 20). In contrast, it was reported that the Nusa Tenggara Barat benefitted from stronger infrastructure, a well-connected network of community health centers or *Puskesmas* and hospitals, and targeted maternal programs, supported by better transportation and higher population density (21, 22). We found that ANC attendance in Papua remained among the lowest, which may be attributed to factors such as rugged terrain, dispersed settlements, and a relatively weak health system (22). In particular, Central Papua, a newly established province, and Highland Papua face additional challenges, including administrative and logistical delays in health system development (23). Furthermore, ongoing geopolitical instability and security concerns related to armed conflict in these regions may further limit access to healthcare. In some areas, the presence of extremist groups and intermittent violence has made it unsafe for residents to leave their homes, leading to reluctance to visit primary health centers or other health facilities. These complex barriers likely contribute to the persistently low ANC coverage observed in these provinces. Similarly, women living in Maluku Province might also face challenges related to its archipelagic geography and high inter-island travel costs (24). Our findings showed that even within eastern Indonesia, ANC use varied notably across provinces, indicating that challenges in access are not uniformly experienced. These intra-regional disparities further emphasize the broader gap between eastern and western Indonesia, where the latter benefits from stronger infrastructure and better maternal health outcomes. Addressing these layered inequalities requires locally tailored strategies that respond to both regional and province-specific needs.

The increased likelihood of mothers from the poorest households not attending ANC services indicates the importance of socioeconomic disparities in maternal healthcare access. This is consistent with findings from previous studies conducted in Indonesia and other low- and middle-income countries (10, 14, 25–27). In Indonesia, antenatal care is officially provided free of charge at both integrated health posts, locally known as *Pos Pelayanan terpadu* or *Posyandu*, and primary-level facilities, mainly on *Puskesmas*, particularly the beneficiaries of the Indonesian National Health Insurance (NHI). Despite the high NHI coverage in Indonesia, the uptake among its beneficiaries when accessing health services was reported to be suboptimal (27, 28). This is consistent with our previous study from Maluku Province, which revealed that less than 15% of NHI beneficiaries used NHI when accessing health services.^1^ Moreover, although ANC services are provided free of charge, other indirect costs, such as transportation, childcare, and lost income due to time off work, could continue to burden low-income households.

Mothers who did not engage in regular health checkups were less likely to use ANC services. This finding aligns with previous studies (29, 30) indicating that the absence of routine interactions with the healthcare system could lead to low awareness, limited familiarity with medical services, and reduced trust in healthcare providers—all of which might discourage ANC attendance (31).

Our study also confirmed the role of maternal knowledge and awareness in ANC uptake, as previously reported (32). We found that mothers with limited knowledge of stunting were also less likely to attend ANC services, potentially due to a lack of understanding of the role of ANC in preventing stunting and promoting child health. A study conducted in West Java, Indonesia, found that improving maternal knowledge significantly increased interest and motivation to use ANC services (11).

Interestingly, mothers without obstetric complications during pregnancy had a reduced likelihood of attending ANC, as reported in previous studies (30, 32, 33). This suggests that women without complications might perceive less need to seek care during pregnancy. Consequently, mothers who did not have any pregnancy complications might perceive their pregnancies as low risk, leading them to underestimate the importance of ANC services. This perception could result in lower ANC attendance, as they might not recognize the role of routine ANC checkups in detecting and preventing potential complications.

Although not explored in this study, cultural values and local wisdom could influence maternal health-seeking behavior in eastern Indonesia, as reported in several qualitative studies (34–36). Traditional beliefs, such as reliance on family elders or traditional birth attendants, could delay or deter ANC visits. Gender roles, trust in modern healthcare, and local norms also shape service uptake. Therefore, further research to understand and integrate these sociocultural factors is essential to improving ANC uptake and ensuring health programs are culturally appropriate (37–40).

The MOR values from our multilevel analysis revealed important insights into the contextual variation in ANC nonattendance across geographic levels. The high MOR in the null model at the province, district, and PSU levels indicated substantial clustering, suggesting that regional and local factors were significantly associated with ANC nonattendance. After adjusting for the significant explanatory variables, the MOR at the provincial level indicated that the explanatory variables largely explained inter-provincial differences. However, MOR remained high at the district level and increased at the PSU level. This shows persistent unexplained variations at more localized levels. These findings suggest that structural, operational, or contextual barriers, such as uneven health facility distribution, staffing shortages, or access issues at the community level, might continue to limit ANC uptake.

### Policy implications

Our findings indicate the need for sustained efforts to improve ANC uptake in eastern Indonesia. Raising community knowledge and awareness of the universal benefits of ANC, regardless of perceived pregnancy-related risk, is essential. Various health promotion strategies have been reported to be effective in improving maternal and child health knowledge, both individual and community-based interventions, such as through home visits (24), group counselling (41), and culturally tailored community outreach (42). Furthermore, health workers should use every contact opportunity to educate women and their families about the importance of early and regular ANC and the services available to support a healthy pregnancy.

Efforts to ensure that ANC is financially accessible are also critical. Policies that reduce the financial burden on pregnant women, such as subsidies, conditional cash transfers, or integration of ANC costs into national health insurance schemes, should be considered (27). Additionally, our findings underscore the importance of strengthening mobile health outreach (e.g., mobile clinics, home visits, or temporary outreach posts) to bring services directly to communities (43, 44). The use of telemedicine services (45), provision of incentives for rural health workers (46, 47), and ensuring reliable transportation support (24) could help overcome geographical and logistical barriers that will improve service accessibility. Future preparedness strategies should also ensure uninterrupted access to essential maternal health services during public health crises such as pandemics. This requires strengthening health system resilience and implementing adaptable service delivery models that could continue operating during future public health emergencies (18, 48). Our study also suggests that efforts to improve ANC utilization must extend beyond individual-level interventions and address health system gaps and service delivery challenges at community and sub-district levels.

These findings highlight the need for regionally tailored health policies to address both inter- and intra-regional disparities in ANC access. Localized approaches are crucial to accommodate the diverse geographical and administrative contexts in eastern Indonesia. Strengthening subnational health systems—particularly in provinces like Central Papua and Maluku is essential. Bridging the development gap between eastern and western Indonesia requires equitable resource allocation and stronger oversight of maternal health programs is important to ensure progress toward national health goals.

### Strengths and limitations of the study

This study used nationally representative data with a large sample size, enabling a robust analysis of the factors associated with ANC non-use in Indonesia. A key strength is its focus on the eastern region, where access to healthcare is limited. The findings highlight regional disparities and context-specific barriers and offer valuable insights for targeted interventions. The use of multilevel modelling also allowed for a comprehensive assessment of provincial-, district-, and individual-level influences on ANC utilization. However, this study had several limitations. Its cross-sectional design precludes causal inference, and the lack of variables on service access, availability, and affordability limits the analysis. It also lacks qualitative insights into maternal beliefs and perceptions of care. Remote areas may be underrepresented, and reliance on self-reported data may introduce a recall bias.

## Conclusions

In conclusion, this study highlights the key factors associated with ANC non-use in eastern Indonesia, where access to care remains limited. ANC nonattendance was associated with regional disparities, socioeconomic status, health-seeking behavior, knowledge of stunting, and perceived pregnancy risks. Additionally, the geographic isolation, economic hardship, and lingering effects of COVID-19 further hindered the community's access to health services. Targeted region-specific strategies, such as mobile health services, telemedicine, transportation support, and community-based education, will help improve uptake. Furthermore, efforts to develop locally tailored policies and health promotion strategies are essential to ensure that no woman is left behind in accessing quality antenatal care, regardless of geography, income, or perceived risk.

## Data Availability

The raw data supporting the conclusions of this article will be made available by the authors, without undue reservation.

## Footnotes

^1^Titaley CR, Ariawan I, Wahyuningsih W, Iwan RF, Malakauseya MLV, Kimberly VD, et al. The use of Indonesia's national health insurance among its beneficiaries in Maluku, one of the largest archipelagic provinces in the country [manuscript under review]. *BMJ Open*. (2025).
