# Supplementary material for: Determinants of non-use of antenatal care services in eastern Indonesia: analysis of the 2023 Indonesia health survey
Source: Front Glob Womens Health. 2025 Aug 18;6:1649276. doi: 10.3389/fgwh.2025.1649276 (PMC12399656; doi:10.3389/fgwh.2025.1649276)
Supplement: Supplementary file 1 [file Datasheet1.docx]

Supplementary Material

**Supplementary Figure 1**. Proportion of women in West Nusa Tenggara Province who did not use antenatal care services during the pregnancy with their infants aged 0–11 months at the time of the interview, the 2023 Indonesia Health Survey

**Supplementary Figure 2**. Proportion of women in East Nusa Tenggara Province who did not use antenatal care services during the pregnancy with their infants aged 0–11 months at the time of the interview, the 2023 Indonesia Health Survey

**Supplementary Figure 3**. Proportion of women in Sulawesi Region who did not use antenatal care services during the pregnancy with their infants aged 0–11 months at the time of the interview, the 2023 Indonesia Health Survey

**Supplementary Figure 4**. Proportion of women in Maluku Region who did not use antenatal care services during the pregnancy with their infants aged 0–11 months at the time of the interview, the 2023 Indonesia Health Survey

**Supplementary Figure 5**. Proportion of women in Papua Region who did not use antenatal care services during the pregnancy with their infants aged 0–11 months at the time of the interview, the 2023 Indonesia Health Survey
